# Impact of health spending on hospitalization rates in Baltic countries: a comparative analysis

**DOI:** 10.1186/s12913-024-11119-4

**Published:** 2024-06-10

**Authors:** Huan Jiang, Alexander Tran, Inese Gobiņa, Janina Petkevičienė, Rainer Reile, Mindaugas Štelemėkas, Ricardas Radisauskas, Shannon Lange, Jürgen Rehm

**Affiliations:** 1 Institute for Mental Health Policy Research, Centre for Addiction and Mental Health, 33 Ursula Franklin Street, T521, Toronto, ON M5S 2S1 Canada; 2 Campbell Family Mental Health Research Institute, Centre for Addiction and Mental Health, 250 College Street, Toronto, ON M5T 1R8 Canada; 3 Dalla Lana School of Public Health, Health Sciences Building, 155 College Street, 6th floor, Toronto, ON M5T 3M7 Canada; 4 Department of Psychiatry, University of Toronto, 250 College Street, 8th floor, Toronto, ON M5T 1R8 Canada; 5 Institute of Medical Science, Faculty of Medicine, University of Toronto, Medical Sciences Building, 1 King’s College Circle, Room 2374, Toronto, ON M5S 1A8 Canada; 6 Program on Substance Abuse, Public Health Agency of Catalonia, 81-95 Roc Boronat St, Barcelona, 08005 Spain; 7 Center for Interdisciplinary Addiction Research (ZIS), Department of Psychiatry and Psychotherapy, University Medical Center Hamburg-Eppendorf (UKE), Martinistraße 52, 20246 Hamburg, Germany; 8 Department of Public Health and Epidemiology, Riga Stradiņš University, Kronvalda Boulevard 9, Riga, LV-1010 Latvia; 9 Health Research Institute, Faculty of Public Health, Lithuanian University of Health Sciences, Tilžės str.18, Kaunas, 47181 Lithuania; 10 Department of Preventive Medicine, Faculty of Public Health, Lithuanian University of Health Sciences, Tilžės str.18, Kaunas, 47181 Lithuania; 11 Department of Epidemiology and Biostatistics, National Institute for Health Development, Paldiski mnt 80, Tallinn, 10617 Estonia; 12 Department of Environmental and Occupational Medicine, Faculty of Public Health, Lithuanian University of Health Sciences, Tilžės str. 18, Kaunas, 47181 Lithuania; 13 Institute of Cardiology, Lithuanian University of Health Sciences, Sukileliu av. 15, Kaunas, 50162 Lithuania

**Keywords:** Health Spending, Hospitalization, Baltic Countries, GAM model, Healthcare systems

## Abstract

**Introduction:**

This study examines the association between healthcare indicators and hospitalization rates in three high-income European countries, namely Estonia, Latvia, and Lithuania, from 2015 to 2020.

**Method:**

We used a sex-stratified generalized additive model (GAM) to investigate the impact of select healthcare indicators on hospitalization rates, adjusted by general economic status—i.e., gross domestic product (GDP) per capita.

**Results:**

Our findings indicate a consistent decline in hospitalization rates over time for all three countries. The proportion of health expenditure spent on hospitals, the number of physicians and nurses, and hospital beds were not statistically significantly associated with hospitalization rates. However, changes in the number of employed medical doctors per 10,000 population were statistically significantly associated with changes of hospitalization rates in the same direction, with the effect being stronger for males. Additionally, higher GDP per capita was associated with increased hospitalization rates for both males and females in all three countries and in all models.

**Conclusions:**

The relationship between healthcare spending and declining hospitalization rates was not statistically significant, suggesting that the healthcare systems may be shifting towards primary care, outpatient care, and on prevention efforts.

**Supplementary Information:**

The online version contains supplementary material available at 10.1186/s12913-024-11119-4.

## Introduction

In the early 1990s, the three Baltic countries, Estonia, Latvia and Lithuania, declared their independence and chose a path that was distinct from the other states by aligning with the goal of joining the European Union (EU). Upon successfully becoming EU members in 2004, their healthcare reforms were predominantly driven by a commitment to establishing a Western-oriented healthcare system, compatible with EU standards [1]. However, despite facing the same major milestones (joining the EU, experiencing an economic crisis between 2008 and 2010, facing the COVID–19 pandemic in 2020), each of the three countries still experienced healthcare reforms that were unique in relation to one another [2, 3].

Within a healthcare system, costs for healthcare services provided by hospitals contribute substantially to overall healthcare costs [4], but their share of overall health expenditure varies by country [4]. Thus, when a healthcare system is being established it must be decided what role hospitals will play—that is, the extent to which they will be used—and this decision has consequences not only for the system itself, but also for population health. For instance, if a country opts for hospitals to play a much smaller role in the treatment and recovery of those who are ill, then the onus ends up being placed on outpatient care facilities, especially on family physicians [5]. Countries with better access to non-hospital healthcare services, measured by health expenditure, or number of overall employed healthcare workers per 10,000 population, may experience lower hospitalization rates, as timely and effective healthcare intervention can prevent or manage health conditions before they become severe.

The relationship between hospitalization rates and hospital-specific indictors such as the number of hospital physicians and nurses or the number of hospital beds is complex and can by influenced by various factors. Roemer’s Law implies that the supply of hospital beds increases hospitalization rates [6]. Some researchers have found evidence of a statistically significant positive relationship between hospital bed availability and patient hospitalization rates [7], while other researchers have reported contrasting results [8–10]. Moreover, improving hospital care without taking into consideration the necessary aftercare in the community post discharge may lead to an increased risk of preventable re-hospitalizations for chronic disease [11].

The association between healthcare indicators and hospitalization rates is further complicated by economic wealth of a country. A higher gross domestic product (GDP) per capita can contribute to better access to medical services, higher health expenditures, and better healthcare infrastructure [12], potentially reducing the overall need for hospitalization. On the other hand, a wealthier population might have higher life expectancy resulting in increases in hospital utilization [13]. Finally, the distribution of wealth within a country may have an effect as well [14].

In the current paper, we sought to evaluate the association between various indicators of healthcare spending and hospitalization rates in the Baltic countries from 2015 to 2020 (i.e., including the first year of the COVID–19 pandemic period). By doing so, we aim to reconcile conflicting results and enhance our comprehension of the indicators influencing hospitalization rates.

We opted to model hospitalization rates by sex because, even though the EU is recognized as having good healthcare coverage for its population [15], universal access to healthcare services has not yet been achieved, and a sex gap in favor of males in access to care has been noted [16, 17]. Surprisingly, despite the lower life expectancy of males, the advantage in life expectancy for females does not translate into a higher number of healthier life years nor in a greater proportion of met medical needs (see also [18]).

## Methods

### Outcome variable: hospitalization rates

A hospitalization is defined as a single inpatient episode chosen based on the type of hospital discharge (i.e., discharged or deceased, but not transferred). All acute, planned, and long-term hospitalizations were included in the analysis. They were calculated as the number of residents from a defined country who were hospitalized, divided by the total population for that country. The original data from Lithuania were in the form of individual entries for hospitalizations with detailed data on patient age, hospital location, ICD codes associated with the hospitalization, and unique patient as well as treatment codes. We collapsed the individual entries based on age and month of hospitalization as well as ICD code [19], resulting in a monthly number of total hospitalizations. This data was further aggregated into quarterly data. In contrast, for Estonian and Latvian data, the total number of quarterly hospitalizations were obtained from their respective databases. We computed hospitalization rates (per 100,000 population) by dividing the number of hospitalizations by population, standardized according to the 2013 European standard population. The hospitalization rate is for patients aged 20 years and older. For Estonia, data for the period from 2015 Q1 to 2020 Q4 was obtained from Estonian Health Insurance Fund’s database and processed by National Institute of Health Development [20]. For Lithuania, data for the period from data from 2015 Q1 to 2019 Q4 was obtained from the compulsory health insurance information system “Sveidra” of the National Health Insurance Fund under the Ministry of Health via the Institute of Hygiene ( [21]; data provided upon request). For Latvia, the data for the period of data from 2015 Q1 to 2020 Q4 was obtained from the National Health Services (NHS) database, via the Healthcare Quality and Monitoring System, processed by the Center for Disease Prevention and Control for Latvia ( [22].

### Independent indicators

#### Hospital-specific indicators

Several hospital-specific indicators were examined, including hospital beds per 1,000 population, hospital percentage share of total current health expenditure, number of physicians and nurses (full-time equivalent) employed in hospitals per 100,000 population. The first indicator came from the Global Burden of Disease (GBD) study 2019 covariates [23], while the latter two were from Eurostat [24] for the years 2015 to 2020. The term ‘hospital beds’ includes beds in both general and specialized hospitals and excludes beds in long-term care facilities. Specifically, it refers to beds that are regularly maintained and staffed that are immediately available for use. Similar to the previous health indicators, all statistics were year- and country-specific.

### Economic indicators

The general economic performance of an individual country was measured by nominal GDP per capita in USD, calculated by dividing the GDP of a country by its population. It was extracted from the Organization for Economic Co-operation and Development (OECD) database for the year 2015 to 2020 [25].

We used population rates for all indicators as described in the following. To improve the readability of the text, we often use an abbreviated form such as medical doctors to describe trends or correlation, but these always refer to the rates listed below.

### Health indicators

The following healthcare indicators were obtained from the GBD Study 2019 covariates [23]: health expenditure per capita (in 2018 USD), number of employed medical doctors per 10,000 population, number of employed health workers (any specialty) per 10,000 population. In addition, life expectancy from the World Bank [26] was included as a measure of public health [27, 28], calculated based on age-specific mortality rates by constructing life tables. All selected indicators were year- and country-specific.

### Statistical analysis

#### Correlation analysis

Hospital specific indicators are influenced by a myriad of factors outside the role of hospitals in a healthcare system, such as GDP per capita, overall health expenditure per capita, overall number of employed medical doctors per 10,000 population, and other factors. In order to examine the associations among the indicators, correlation analysis was applied, and Pearson’s correlation coefficient was used to compare these continuous variables. Indicators with high correlations were excluded from the subsequent modelling process, as highly correlated variables violate the assumptions of the statistical models employed. These correlated variables also do not provide additional information, and only introduce complexity into subsequent analyses. The rationale behind variable selection is further elucidated in the Results section.

#### Statistical models

To test our hypothesis that selected health indicators had an impact on hospitalization rates in the Baltic countries, we applied a generalized additive model (GAM) for both males and females for each indicator, adjusting for GDP per capita [29]. The mathematical form of the GAM model is provided in the Appendix. All three countries were included in the analyses and were represented by a categorical variable with Latvia, the country with lowest average GDP per capita, serving as the reference category. That is, the coefficients of country effects should be interpreted with respect to Latvia. The standardized hospitalization rates were approximately normally distributed after log transformation, allowing for the use of linear models. Seasonality was adjusted for by adding smoothing splines representing the quarterly patterns. To examine the historical trends and the impacts of general economic performance, we presented four models, each of which included one health indictor of interest, adjusted for by country and the interactions between time and country, in addition to GDP per capita and the smooth terms. Akaike information criterion (AIC) and R-squared values were used to assist in evaluating the fitness of models [30]. The analyses were conducted separately for each sex, resulting in four models for each. Since the analyses incorporated data from the year 2020, a period impacted by the COVID–19 pandemic with potential influences on hospital utilization, a sensitive analysis was conducted using only the data from the years 2015 to 2019. It is important to note that indicators from the GBD database are available only up to the year 2019; in such cases, the sensitivity analyses mirrored the main analyses.

All analyses were performed using R version 4.2.1 [31].

## Results

**Fig. 1:**
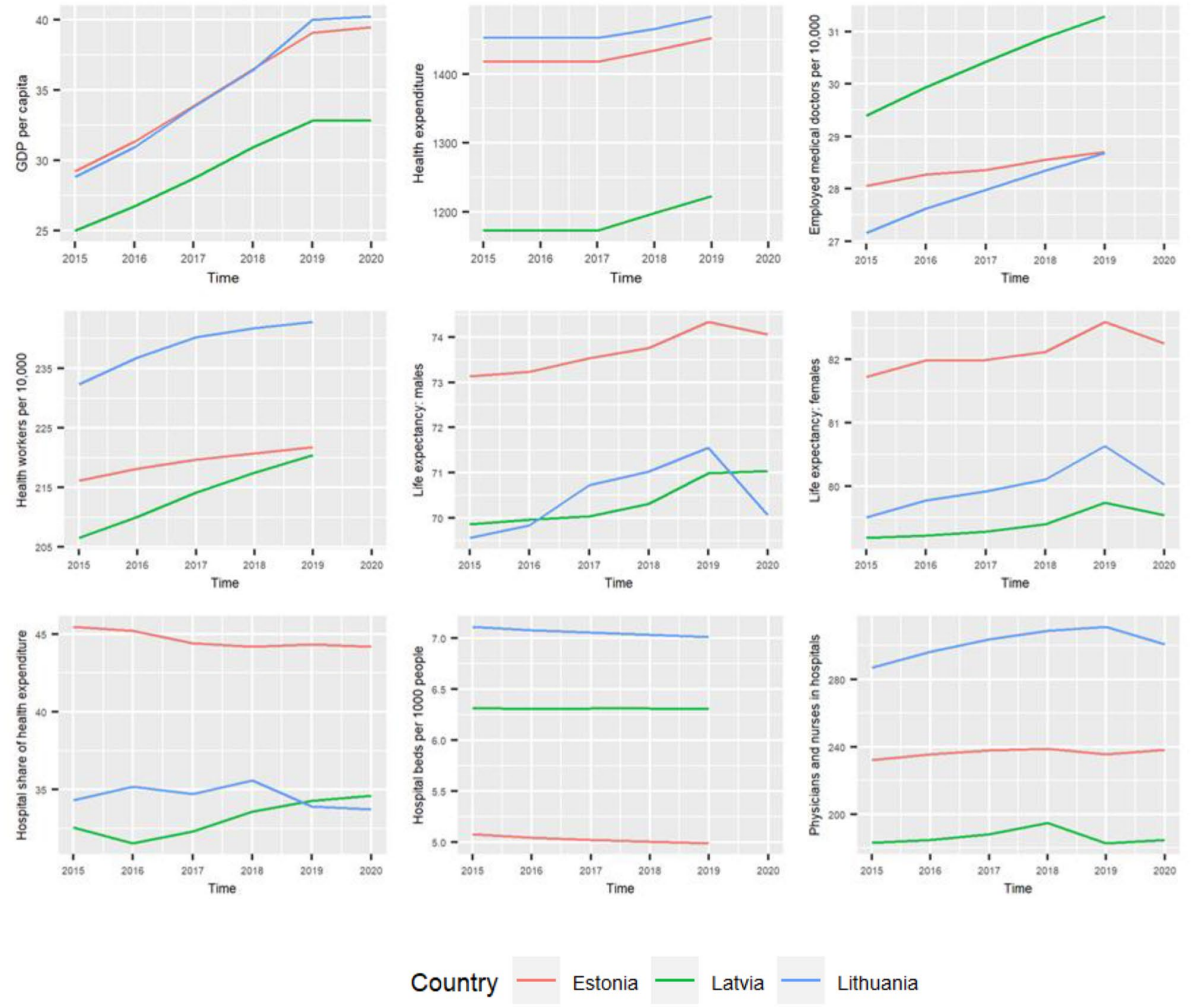
Temporal trends of selected health indicators in the Baltic countries, 2015–2020, including GDP per capita, health expenditure per capita (in 2018 USD), number of employed medical doctors per 10,000 population, number of employed health workers (any specialty) per 10,000 population, life expectancy for males, life expectancy for females, hospital percentage share of total current health expenditure, hospital beds per 1,000 population, number of physicians and nurses (full-time equivalent) employed in hospitals per 100,000 population. Source: data from the World Bank 2015–2020, Eurostat 2015–2020 and Global Burden of Disease (GBD) Study 2015–2019 covariates

Figure 1 illustrates a general upward trend in most indicators over time. However, some hospital-specific indicators, such as the number of hospital beds and hospital share of health expenditure, remained relatively constant or decreased slightly during the study period.

Life expectancies for both males and females were highly correlated (Table A1 in Appendix) with each other (Pearson’s *R* = 0.98, *p* < 0.01). Additionally, there was a notable negative correlation with the number of hospital beds per 1,000 population for both sexes (Pearson’s *R* = -0.85 and − 0.81, *p* < 0.01 and *p* < 0.01), and a positive correlation with the hospital percentage shares of total current health expenditure (Pearson’s *R* = 0.94 and 0.96, *p* < 0.01 and *p* < 0.01). Therefore, as our primary interest was in hospital-related indicators, life expectancies were excluded from subsequent analyses. Similarly, the numbers of employed health workers (any specialty) per 10,000 population showed a high correlation with the number of physicians and nurses (full-time equivalent) employed in hospitals per 100,000 population (Pearson’s *R* = 0.94, *p* < 0.01), leading to their exclusion from further statistical modelling. Consequently, the number of physicians and nurses (full-time equivalent) employed in hospitals per 100,000 population and the three hospital-specific indicators were investigated in the subsequent GAM models after adjusting for GDP per capita.

**Fig. 2:**
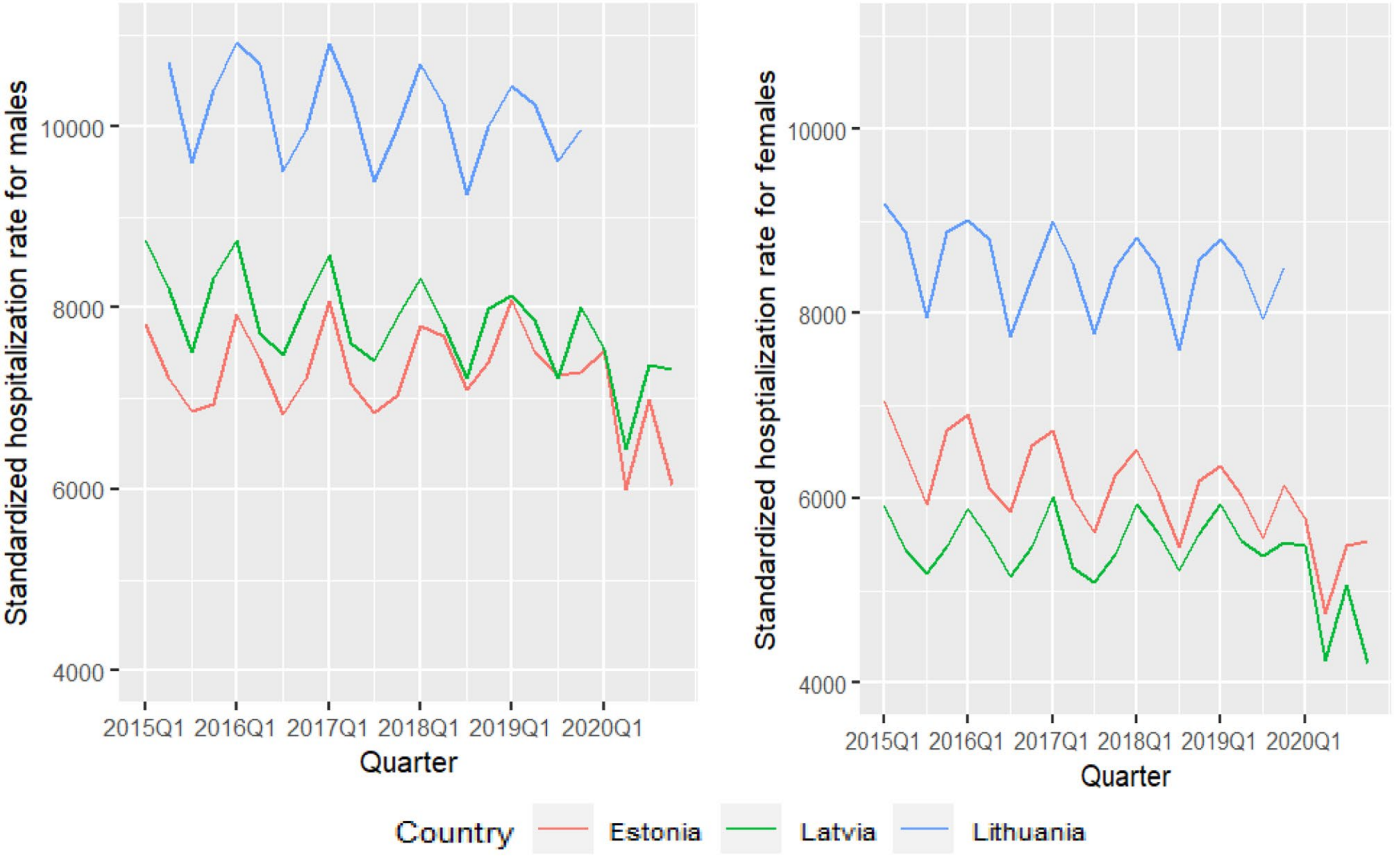
Hospitalization rates (per 100,000 people), age-standardized according to the European standard [1], 2015 q1-2020 q4. Source: Authors’ calculations based on data from Estonia, Latvia, and Lithuania. See data sources for details

Over the study period, standardized hospitalization rates decreased in all three countries (Fig. 2). It is noteworthy that the rates experienced a sudden decrease since the start of the pandemic, around March 2020, in Latvia and Estonia. The figure also depicts seasonal complexities of a quarterly pattern. To separate time trends and quarterly fluctuations, we introduced cubic smoothing spline functions in the GAM models. Tables 1 and 2 showed the results of the male and female GAM models using 2015 to 2020 data, and the sensitivity analysis using 2015–2019 data. The estimates of the smooth functions and examples of model diagnoses can be found in the Appendices 2–4. In all models and for both sexes, the decline in hospitalization rates showed statistical significance over time. Furthermore, the impact of GDP per capita on hospitalization rates exhibited significance across all models, with coefficients ranging from 0.02 to 0.04 in $1,000 (Tables 1 and 2); reflecting that for every $1,000 increase in GDP per capita, the hospitalization rates increased by 2–4%. As the interaction effects of country and time were statistically significant in the majority of models, it can be concluded that the historical trends in countries generally exhibited a downward trend, but with variations between countries over time.

For males (Table 1), after adjusting by GDP per capita, the number of hospital beds was not statistically related to the hospitalization rates (*p* = 0.37), nor was it for the hospital share of health expenditure (*p* = 0.22) or for the number of doctors and nurses employed in hospitals (*p* = 0.48). That is, there were no statistically significant associations between any of the hospital indicators and the hospitalization rates. However, the overall number of employed medical doctors per 10,000 population was significantly related to the hospitalization rates (*p* < 0.001): every unit of increase in employed medical doctors related to a 16% increase in hospitalization rate (exp(0.15)-1).

Slightly different from what was observed for males, after adjusting for GDP per capita, the number of hospital beds was marginally related to the hospitalization rates in females (*p* = 0.07; Table 2), which indicated a 213.93% (exp(1.14)-1) increase for each unit of increase in the number of hospital beds per 1,000 population. In addition, the number of employed medical doctors per 10,000 population was not statistically significantly related to the outcome for females (*p* = 0.09).

The sensitivity analyses used the same models with data from the years 2015 to 2019 exclusively (Tables 1 and 2, right panel). The findings consistently support those derived from the main analyses.

**Table 1:** Effect of various indictors on hospitalization rates for males

	Using 2015–2020 data				Using 2015–2019 data			
	Estimate	Std. Error	Confidence Interval	p-value	Estimate	Std. Error	Confidence Interval	p-value
Model 1	R-square = 0.975				Same			
Intercept	3.911	4.585	(-5.076, 12.898)	0.398				
Time	-0.017	0.003	(-0.023, -0.011)	< 0.001				
Estonia	0.763	0.861	(-0.925, 2.451)	0.38				
Lithuania	-0.269	0.586	(-1.418, 0.880)	0.649				
GDP	0.039	0.006	(0.027, 0.051)	< 0.001				
Hospital Bed	0.641	0.711	(-0.753, 2.035)	0.372				
Time.Estonia**	-0.007	0.003	(-0.013, -0.001)	0.046				
Time.Lithuania	-0.008	0.004	(-0.016, -0.000)	0.021				
Model 2	R-square = 0.939				R-square = 0.976			
Intercept	7.467	0.370	(6.742, 8.192)	< 0.001	7.728	0.230	(7.277, 8.179)	< 0.001
Time	-0.023	0.003	(-0.029, -0.017)	< 0.001	-0.017	0.003	(-0.023, -0.011)	< 0.001
Estonia	-0.241	0.148	(-0.531, 0.049)	0.109	-0.149	0.090	(-0.325, 0.027)	0.106
Lithuania	0.176	0.047	(0.084, 0.268)	< 0.001	0.225	0.030	(0.166, 0.284)	< 0.001
GDP	0.044	0.006	(0.032, 0.056)	< 0.001	0.036	0.004	(0.028, 0.044)	< 0.001
Hospital Share	0.012	0.010	(-0.008, 0.032)	0.22	0.009	0.006	(-0.003, 0.021)	0.128
Time. Estonia	-0.005	0.003	(-0.011, 0.001)	0.048	-0.007	0.002	(-0.011, -0.003)	< 0.001
Time. Lithuania	-0.009	0.003	(-0.015, -0.003)	0.001	-0.01	0.002	(-0.014, -0.006)	< 0.001
Model 3	R-square = 0.723				R-square = 0.854			
Intercept	7.693	0.307	(7.091, 8.295)	< 0.001	7.961	0.205	(7.559, 8.363)	< 0.001
Time	-0.021	0.003	(-0.027, -0.015)	< 0.001	-0.016	0.002	(-0.020, -0.012)	< 0.001
Estonia	-0.114	0.080	(-0.271, 0.043)	0.157	-0.036	0.053	(-0.140, 0.068)	0.499
Lithuania	0.107	0.161	(-0.209, 0.423)	0.509	0.212	0.103	(0.010, 0.414)	0.045
GDP	0.042	0.006	(0.030, 0.054)	< 0.001	0.035	0.005	(0.025, 0.045)	< 0.001
Hospital doctor and nurse	0.001	0.002	(-0.003, 0.005)	0.475	0.000	0.001	(-0.002, 0.002)	0.637
Time. Estonia	-0.008	0.002	(-0.012, -0.004)	< 0.001	-0.009	0.001	(-0.011, -0.007)	< 0.001
Time. Lithuania	-0.012	0.003	(-0.018, -0.006)	< 0.001	-0.012	0.002	(-0.016, -0.008)	< 0.001
Model 4	R-square = 0.980							
Intercept	3.969	1.059	(1.893, 6.045)	< 0.001				
Time	-0.025	0.003	(-0.031, -0.019)	< 0.001				
Estonia	0.234	0.067	(0.103, 0.365)	0.001				
Lithuania	0.646	0.102	(0.446, 0.846)	< 0.001				
GDP	0.019	0.006	(0.007, 0.031)	0.002				
Medical doctor	0.153	0.040	(0.075, 0.231)	< 0.001				
Time. Estonia	0.004	0.004	(-0.004, 0.012)	0.245				
Time. Lithuania	-0.005	0.002	(-0.009, -0.001)	0.034				

** It means the interaction between time and country, reflecting distinct trends for various countries

Note: “Hospital bed” refers to the numbers of hospital beds per 1,000 population; “Hospital share” refers to the hospital percentage share of total current health expenditure; “Hospital doctor and nurse” refers to number of physicians and nurses (full-time equivalent) employed in hospitals per 100,000 population; “Medical doctor” refers to the number of employed medical doctors per 10,000 population

**Table 2:** Effect of various indictors on hospitalization rates for females

	Using 2015–2020 data				Using 2015–2019 data			
	Estimate	Std. Error	Confidence Interval	p-value	Estimate	Std. Error	Confidence Interval	p-value
Model 1	R-square = 0.989				Same			
Intercept	0.591	3.911	(-7.075, 8.257)	0.880				
Time	-0.015	0.002	(-0.019, -0.011)	< 0.001				
Estonia	1.463	0.735	(0.022, 2.904)	0.052				
Lithuania	-0.567	0.500	(-1.547, 0.413)	0.262				
GDP	0.033	0.005	(0.023, 0.043)	< 0.001				
Hospital Bed	1.144	0.607	(-0.046, 2.334)	0.065				
Time.Estonia**	-0.005	0.003	(-0.011, 0.001)	0.083				
Time.Lithuania	-0.003	0.003	(-0.009, 0.003)	0.332				
Model 2	R-square = 0.954				R-square = 0.989			
Intercept	7.265	0.418	(6.446, 8.084)	< 0.001	7.701	0.202	(7.305, 8.097)	< 0.001
Time	-0.025	0.004	(-0.033, -0.017)	< 0.001	-0.014	0.002	(-0.018, -0.010)	< 0.001
Estonia	-0.166	0.167	(-0.493, 0.161)	0.324	-0.036	0.079	(-0.191, 0.119)	0.652
Lithuania	0.263	0.053	(0.159, 0.367)	< 0.001	0.346	0.027	(0.293, 0.399)	< 0.001
GDP	0.043	0.007	(0.029, 0.057)	< 0.001	0.028	0.004	(0.020, 0.036)	< 0.001
Hospital Share	0.011	0.011	(-0.011, 0.033)	0.33	0.008	0.005	(-0.002, 0.018)	0.145
Time. Estonia	-0.006	0.003	(-0.012, -0.000)	0.048	-0.008	0.002	(-0.012, -0.004)	< 0.001
Time. Lithuania	-0.006	0.003	(-0.012, -0.000)	0.041	-0.007	0.002	(-0.011, -0.003)	< 0.001
Model 3	R-square = 0.801				R-square = 0.937			
Intercept	7.509	0.346	(6.831, 8.187)	< 0.001	8.032	0.18	(7.679, 8.385)	< 0.001
Time	-0.023	0.003	(-0.029, -0.017)	< 0.001	-0.013	0.002	(-0.017, -0.009)	< 0.001
Estonia	-0.042	0.090	(-0.218, 0.134)	0.641	0.096	0.046	(0.006, 0.186)	0.044
Lithuania	0.225	0.182	(-0.132, 0.582)	0.222	0.414	0.09	(0.238, 0.590)	< 0.001
GDP	0.041	0.007	(0.027, 0.055)	< 0.001	0.027	0.004	(0.019, 0.035)	< 0.001
Hospital doctor and nurse	0.001	0.002	(-0.003, 0.005)	0.662	0	0.001	(-0.002, 0.002)	0.662
Time. Estonia	-0.008	0.002	(-0.012, -0.004)	< 0.001	-0.009	0.001	(-0.011, -0.007)	< 0.001
Time. Lithuania	-0.009	0.004	(-0.017, -0.001)	0.016	-0.008	0.002	(-0.012, -0.004)	< 0.001
Model 4	R-square = 0.989				Same			
Intercept	6.209	1.027	(4.196, 8.222)	< 0.001				
Time	-0.017	0.003	(-0.023, -0.011)	< 0.001				
Estonia	0.184	0.065	(0.057, 0.311)	0.007				
Lithuania	0.542	0.099	(0.348, 0.736)	< 0.001				
GDP	0.02	0.006	(0.008, 0.032)	0.001				
Medical doctor	0.066	0.038	(-0.008, 0.140)	0.092				
Time. Estonia	-0.004	0.004	(-0.012, 0.004)	0.317				
Time. Lithuania	-0.005	0.002	(-0.009, -0.001)	0.016				

** It means the interaction between time and country, reflecting distinct trends for various countries

Note: “Hospital bed” refers to the numbers of hospital beds per 1,000 population; “Hospital share” refers to the hospital percentage share of total current health expenditure; “Hospital doctor and nurse” refers to number of physicians and nurses (full-time equivalent) employed in hospitals per 100,000 population; “Medical doctor” refers to the number of employed medical doctors per 10,000 population

## Discussion

The study found that standardized hospitalization rates decreased in all three Baltic countries, with no statistically significant associations identified with any of the available hospital indicators. The decreasing hospitalization rates in the Baltic countries over the years can be attributed to the policy trend to increase the capacity of ambulatory services (e.g. a day surgery services etc.), the push for greater efficiency, as well as a shift towards providing a broader range of outpatient services [32–34]. The results suggest that healthcare systems develop with similar trends but at different speeds in the three Baltic countries, where hospitalization rates declined, a trend which is typical for most countries of the EU [35].

Even though the utilization of hospitals is complex and influenced by various factors, we found that GDP per capita was significantly and consistently associated with hospitalization rates in all models. Despite the assumption that higher GDP per capita might provide better access to healthcare services, thereby reducing the need for hospitalization, higher GDP per capita was associated with a higher hospitalization rate in those countries. This result was somewhat surprising, as there had been evidence that in aging workforces such as in Europe, negative effects on labor productivity have been observed [36].

Lithuania seems to have a higher hospital bed capacity and a higher number of healthcare workers compared to other EU countries, while the hospital bed capacity in Latvia and Estonia was around the EU average (4.41 hospital beds per 1,000 population in 2020) or even lower. However, this does not mean that life expectancy was the highest in Lithuania. In fact, Estonia has had the highest life expectancy of the three Baltic countries since the early years of the 21st century [26], which may indicate that their overall healthcare system is better than the other two (but healthcare systems only contribute to a proportion of life expectancy [37]).

The interrelationships between the health indicators investigated here were similar in all three Baltic countries. Firstly, hospitalization rates were not significantly associated with hospital-specific indicators, including hospital beds, hospital percentage of health expenditures, and numbers of physicians and nurses working in hospitals. More interestingly, there seems to be a very strong relationship between the overall number of employed medical doctors per 10,000 population and hospitalization rates. In fact, given the economic pressure to reduce costs, the number of hospital physicians and nurses did not increase, but the number of medical doctors in healthcare systems did increase in the Baltic countries over time (see Fig. 1). Given the overall aging and shrinking Baltic populations [38], there has been a shift towards primary care and preventive measures in all three countries [33].

The association between the number of hospital beds and hospitalization rates was more pronounced for females than for males, which may be tied to the fact that a greater proportion of females report experiencing unmet healthcare needs [39]. If patients’ healthcare needs are not met, their conditions may be more severe when they present to hospital, and they may require re-admission, potentially increasing the number of hospital admissions.

We would like to point out some potential strengths and limitations to this study. First, our analyses were based on routine data, which are usually without much bias, as hospitalization rates, number of hospital beds, and overall health expenditures can be measured relatively easily. Underlying definitions used for calculating the rates of hospitalizations were similar in the collection of data, and for definitions of hospital beds and overall health expenditures, use of the GBD study data for covariates ensured that comparable definitions were used as well [40]. The same applies for all the other covariates. One potential limitation concerns the specification of the statistical model. Of course, we cannot guarantee that we did not miss important covariates, but the overall goodness of fit with more than 95% of variance explained in both reduced models could be taken as a good indicator that our variables were highly predictive. Another limitation is that the overall sample size was relatively small for the type of statistical analyses selected [29]. However, since the main effects had low p-values and the overall proportion of variance explained was high, we do not see this as a major limitation.

In summary, we observed variations in hospitalization rates among the Baltic countries, although there appears to be some convergence in more recent years. However, the relationships between various hospital-specific indicators and hospitalization rates were not statistically significant, suggesting that the healthcare systems may be shifting towards primary care, outpatient care, and on prevention efforts. The impact of GDP per capita on hospitalization rates should be further explored. There seem to be different underlying processes: while the impact of aging populations, which are typical for high income countries, would predict higher hospitalization rates and negative impacts on GDP per capita, other processes such as higher technological progression may outweigh these population factors. In addition, the relationship between the overall number of employed medical doctors and hospitalization rates requires additional clarification. Notably, we observed a more pronounced association among males compared to females, despite a greater proportion of females report experiencing unmet healthcare needs [39]. Thus, future investigations could explore gender disparities and formulating policies aimed at addressing this difference.

### Electronic supplementary material

Below is the link to the electronic supplementary material.

Supplementary Material 1

## Data Availability

The datasets used and/or analyzed during the current study are available from the corresponding author on reasonable request.
