# Supplementary material for: Impact of health spending on hospitalization rates in Baltic countries: a comparative analysis
Source: BMC Health Serv Res. 2024 Jun 10;24:714. doi: 10.1186/s12913-024-11119-4 (PMC11165763; doi:10.1186/s12913-024-11119-4)

# Appendix

**1 Generalized Addictive Model (GAM)**

A significant advantage of GAM is its capability to analyze non-linear relationships while adjusting for potential covariates. Nevertheless, GAMs require that errors between observations are independent, a condition that is challenging in time series data where autocorrelation is a common feature.

$$\begin{matrix} Y_{i}\sim exp\left( \mu_{i},.. \right) \\ E\left( Y_{i} \right)=\mu_{i} \\ g\left( \mu_{i} \right)=\eta_{i}=X_{i}\beta_{i}+ {f(s}_{j}) \end{matrix}$$

where the response variable $Y_{i}$ follows an exponential family distribution. $g$ is a monotonic link function. $X_{i}$ is the independent variable, such as time, GDP per capita and hospital specific indicators. $f_{j}$ are the smooth functions of the covariates $s_{j}$, the seasonal trend. Penalized Iteratively Reweighted Least Squares are used as the iterative algorithm that estimates the parameters of the model, when penalty is applied to some parameters for smoothness.

**2 Correlations Among Various Economics and Health Indicators**

**Table A1**

**Correlations Among Various Economics and Health Indicators: including GDP per capita, health expenditure per capita (in 2018 USD), number of employed medical doctors per 10,000 population, number of employed health workers (any specialty) per 10,000 population, life expectancy for males, life expectancy for females, hospital percentage share of total current health expenditure, hospital beds per 1,000 population, number of physicians and nurses (full-time equivalent) employed in hospitals per 100,000 population**

| Indicators | GDP per capita | Health expenditure | Medical doctors | Health workers | Life expectant: males | Life expectant: females | Hospital share | Hospital beds | Physicians and nurses in hospitals |
| --- | --- | --- | --- | --- | --- | --- | --- | --- | --- |
| GDP per capita |  |  |  |  |  |  |  |  |  |
| Health expenditure | 0.66*** |  |  |  |  |  |  |  |  |
| Medical doctors | -0.19 | -0.85*** |  |  |  |  |  |  |  |
| Health workers | 0.60*** | 0.74*** | -0.52*** |  |  |  |  |  |  |
| Life expectant: males | 0.63*** | 0.48*** | -0.23 | -0.11 |  |  |  |  |  |
| Life expectant: females | 0.61*** | 0.59*** | -0.39** | -0.02 | 0.98*** |  |  |  |  |
| Hospital share | 0.43*** | 0.53*** | -0.41** | -0.13 | 0.94*** | 0.96*** |  |  |  |
| hospital beds | -0.11 | -0.04 | -0.02 | 0.59*** | -0.85*** | -0.81*** | -0.86*** |  |  |
| Physicians and nurses in hospitals | 0.47*** | 0.87*** | -0.77*** | 0.94*** | 0.04 | 0.19 | 0.10 | 0.43*** |  |

**SOURCES**: Authors’ calculations based on data from the World Bank, Eurostat and Global Burden of Disease (GBD) Study 2015-2019 covariates. **NOTE**: *** means P-value less than 0.001.

**3 Seasonal effects**

### Smooth functions for men’ models


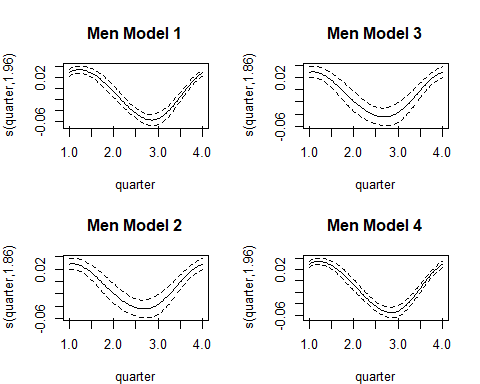


### Smooth functions for women’ models


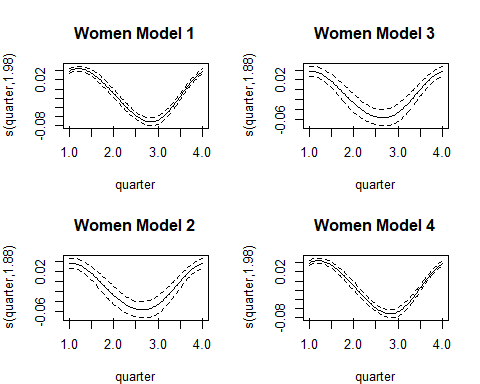


### 4 Diagnostics examples

### Diagnostics of fitted GAM model 1 for males


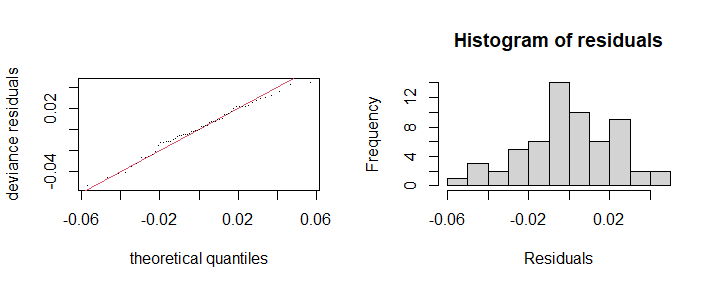

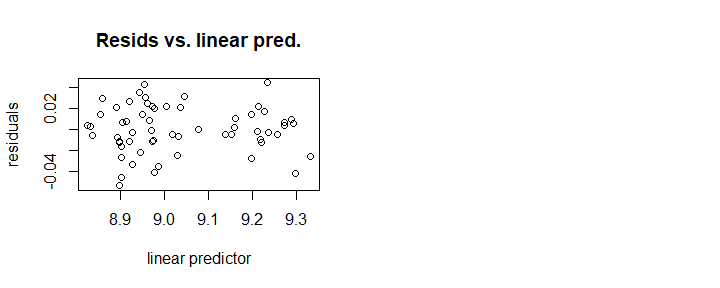


### Diagnostics of fitted GAM model 1 for females


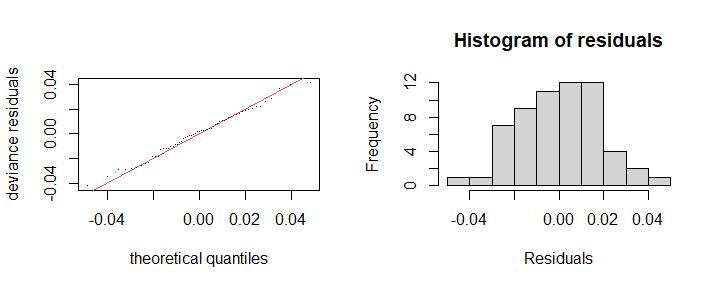

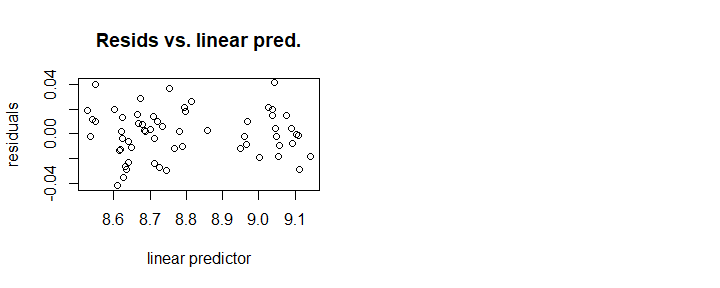

Supplement: Supplementary file 1 — Supplementary Material 1 [file 12913_2024_11119_MOESM1_ESM.docx]
